# Objective assessment of the effects of opicapone in Parkinson’s disease through kinematic analysis

**DOI:** 10.1007/s10072-023-07233-6

**Published:** 2023-12-13

**Authors:** Matteo Bologna, Andrea Guerra, Donato Colella, Daniele Birreci, Davide Costa, Antonio Cannavacciuolo, Luca Angelini, Giulia Paparella, Angelo Antonini, Alfredo Berardelli, Giovanni Fabbrini

**Affiliations:** 1 Department of Human Neurosciences, Sapienza University of Rome, Viale Dell’Università 30, 00185 Rome, Italy; 2 IRCCS Neuromed, 86077 Pozzilli, IS Italy; 3 Parkinson and Movement Disorder Unit, Study Center On Neurodegeneration (CESNE), Department of Neuroscience, University of Padua, Padua, Italy; 4 Present Address: Padua Neuroscience Center, University of Padua, Padua, Italy

**Keywords:** Parkinson’s disease, Bradykinesia, L-DOPA, Opicapone, Kinematic analysis

## Abstract

**Background:**

Opicapone (OPC) is a third-generation, selective peripheral COMT inhibitor that improves peripheral L-DOPA bioavailability and reduces OFF time and end-of-dose motor fluctuations in Parkinson’s disease (PD) patients.

**Objectives:**

In this study, we objectively assessed the effects of adding OPC to L-DOPA on bradykinesia in PD through kinematic analysis of finger movements.

**Methods:**

We enrolled 20 treated patients with PD and motor fluctuations. Patients underwent two experimental sessions (L-DOPA, L-DOPA + OPC), separated by at least 1 week. In each session, patients were clinically evaluated and underwent kinematic movement analysis of repetitive finger movements at four time points: (i) before their usual morning dose of L-DOPA (T0), (ii) 30 min (T1), (iii) 1 h and 30 min (T2), and (iv) 3 h and 30 min after the L-DOPA intake (T3).

**Results:**

Movement velocity and amplitude of finger movements were higher in PD patients during the session with OPC compared to the session without OPC at all the time points tested. Importantly, the variability of finger movement velocity and amplitude across T0–T3 was significantly lower in the L-DOPA + OPC than L-DOPA session.

**Conclusions:**

This study is the first objective assessment of the effects of adding OPC to L-DOPA on bradykinesia in patients with PD and motor fluctuations. OPC, in addition to the standard dopaminergic therapy, leads to significant improvements in bradykinesia during clinically relevant periods associated with peripheral L-DOPA dynamics, i.e., the OFF state in the morning, delayed-ON, and wearing-OFF periods.

**Supplementary Information:**

The online version contains supplementary material available at 10.1007/s10072-023-07233-6.

## Introduction

Advanced stage Parkinson’s disease (PD) is characterized by shortening of L-DOPA efficacy and appearance of motor complications including wearing-off phenomena [1]. Several strategies can be employed to minimize the duration and severity of OFF time and related bradykinesia and extend the duration of the therapeutic effects of L-DOPA [2]. Opicapone (OPC), a third-generation and selective peripheral catechol O-methyl transferase (COMT) inhibitor, plays a crucial role in the catabolism of L-DOPA and alleviates response fluctuations that occur towards the end of each L-DOPA dose [3, 4]. Clinical trials have demonstrated that OPC is not inferior to its predecessor, entacapone, when maintaining a stable dopaminergic response. However, OPC enables once-daily dosing, and its primary advantage over entacapone becomes evident especially in patients presenting predictable wearing-off periods [5]. Despite various clinical trials confirming OPC’s potential through subjective clinical assessments, there are no specific and objective studies documenting the improvement of bradykinesia after OPC intake using kinematic analysis.

Kinematic techniques, albeit with methodological heterogeneity, have been used to quantitatively assess bradykinesia and the effects of various medications on motor performance in PD [6–10]. In this regard, kinematic analysis of finger tapping, which includes the repetitive opposition movement of the thumb and index finger over time, is one of the most valuable approaches for assessing bradykinesia and evaluating the impact of dopaminergic replacement in PD [7, 9, 11]. Several studies have demonstrated that bradykinesia features include decreased voluntary movement velocity as well as reduced movement amplitude (hypokinesia) and a decline in both amplitude and speed during repetitive movement (sequence effect) [7, 12, 13]. These features may vary in their presence across PD patients, and they might exhibit different sensitivity to changes in response to dopamine replacement therapy [8, 13].

In this study, our objective was to evaluate the efficacy of adding OPC, on the various bradykinesia features using clinical and kinematic analysis. We examined the impact of OPC on finger movements in PD patients at various time intervals throughout the day following L-DOPA administration, compared to patients treated with L-DOPA only.

## Methods

### Participants

The study was conducted at the Department of Human Neurosciences, Sapienza University of Rome. Participant recruitment took place from September 2020 to October 2022. We enrolled 20 PD patients with a clinical diagnosis based on current diagnostic criteria [14]. All patients experienced wearing-off phenomena after ≤ 3.5 h from the intake of L-DOPA. The main exclusion criteria included symptoms and signs suggestive of atypical parkinsonism, exposure to medications or substances affecting the central nervous system that are not part of PD therapy, patients with moderate-to-severe cognitive impairment, or additional neuropsychiatric conditions. The patients were clinically evaluated using standardized clinical scales, including the Movement Disorder Society-Sponsored Revision of the Unified Parkinson’s Disease Rating Scale (MDS-UPDRS-II, MDS-UPDRS-III, MDS-UPDRS-IV) [15–17], the Montreal Cognitive Assessment (MoCA) [18], and Beck Depression Inventory (BDI-II) [19]. A group of 20 age- and gender-matched healthy controls (HCs; 67.0 ± 5.4 years, 7 females) was also recruited. All participants provided informed consent to the experimental procedures, which were approved by the local ethics committee. The study was conducted in compliance with the Declaration of Helsinki.

### Kinematic analysis

After ensuring a comfortable position, all PD patients were asked to perform repetitive thumb–index opposition movements (finger tapping) with the upper limbs for three consecutive 15-s trials. Each participant was given a practice trial before the actual execution, and a 1-min break was provided between each series to avoid fatigue. Three series of finger tapping were performed for each of the four time points in the two experimental sessions (without OPC and with OPC). HCs were evaluated in a single experimental session, and the kinematic recordings of repetitive finger movements (of the dominant hand) were carried out in the same manner as the PD patients. The examiners consistently encouraged all participants to perform their movements in the fastest and widest possible way in each experimental session.

Finger tapping movements were examined using an optoelectronic system for three-dimensional (3D) motion analysis (SMART DX 100, BTS, Milan, Italy). This system comprises three infrared cameras (sampling frequency 120 Hz) that record the three-dimensional displacement of reflective markers, which are lightweight and placed on the body segments under examination. Reflective markers were positioned as follows: two markers were placed on the distal phalanges of the first and second fingers, and the remaining four markers were placed on the head and base of the second metacarpal, the base of the fifth metacarpal, and the wrist, to establish a reference plane on the hand and exclude any contamination of finger movements due to undesired hand movements [7, 12, 20]. The analysis of kinematic data was performed using dedicated software (SMART Analyzer, BTS, Milan, Italy) to calculate the kinetic variables of interest, including the number of movements, amplitude, and velocity of the movement expressed in degrees (°) and degrees per second (°/s). Additionally, the decrement in amplitude and velocity during movement repetition and the coefficient of variation (CV), an expression of rhythm defined as the ratio of standard deviation (SD) to the mean value of intervals in three-tapping, were calculated, with higher values indicating less rhythmic movement [7, 12, 20, 21].

### Experimental procedure

**Fig. 1:**
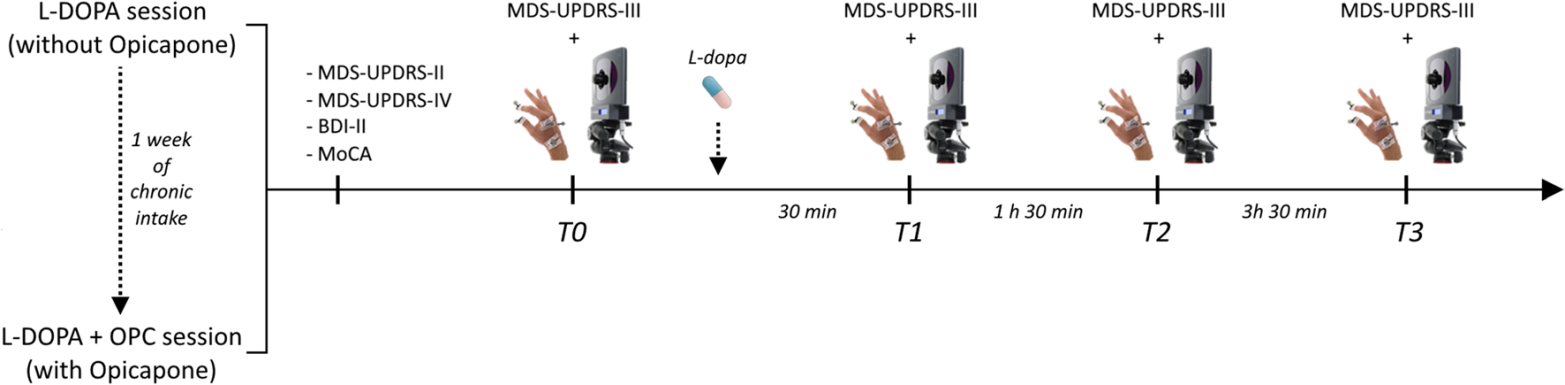
Experimental design. PD patients underwent two experimental sessions: (i) L-DOPA and (ii) L-DOPA + OPC. In each session, we performed motor and non-motor clinical scales and kinematic evaluations of finger tapping movements at four distinct time points

The study comprised two sessions: (i) a session without OPC, during which patients continued their regular dopaminergic therapy (L-DOPA), and (ii) a session after at least 1 week of daily intake of OPC 50 mg/day as an adjunct treatment to their usual dopaminergic therapy (L-DOPA + OPC). Our choice of this particular timeframe stems from prior pharmacodynamic studies that consistently revealed a significant and enduring inhibition of COMT activity within the initial 7 days of initiating OPC therapy [22, 23]. Notably, there were no alterations in the drug regimen between session I and II, with the sole exception of the addition of OPC. Each session involved both clinical (MDS-UPDRS-III) and kinematic evaluations at four distinct time points, determined by the time elapsed since the administration of the first daily dose of L-DOPA: (i) prior to taking the first L-DOPA dose in the morning (T0), (ii) 30 min after L-DOPA intake (T1), (iii) 1 h and 30 min after L-DOPA intake (T2), and (iv) 3 h and 30 min after L-DOPA intake (T3) (Fig. 1). These specific time points were chosen to capture clinically relevant periods associated with L-DOPA dynamics and motor fluctuations. Specifically, T0 reflected the OFF dopaminergic condition in the morning, T1 captured potential delayed-ON phenomena, T2 coincided with the peak effect of L-DOPA (i.e., ON dopaminergic condition), and T3 represented the timing of wearing-off phenomena, as reported by our patients (see “Participants”).

At the beginning of each session, we also evaluated the motor aspects of experiences of daily living (MDS-UPDRS-II), motor complications severity (MDS-UPDRS-IV) referred to the previous week of treatment, cognition (MoCA) [18], and depressive symptoms using the Beck Depression Inventory (BDI-II) [19] (Fig. 1). Importantly, the researcher who assessed MDS-UPDRS-II, III, and IV was blinded to the experimental session.

### Statistical analysis

The sample size calculation was performed using the G*Power software. We set a desired power of 0.80 and an alpha error of 0.05, assuming a 20% change in kinematic measures between PD and HCs based on previous studies [7, 11, 24]. A sample size of 18 participants was required to detect a significant difference between groups. The Fisher’s exact test was used to assess gender differences between PD patients and HCs, while the Mann–Whitney *U* test was adopted to compare age and clinical scores between the two groups. Possible differences in the clinical scales in patients between the two experimental sessions were tested using the Wilcoxon test. Also, two separate Friedman analyses of variance (ANOVAs) were adopted to verify possible changes in the MDS-UPDRS-III between the various time points in the L-DOPA and L-DOPA + OPC sessions. We computed both the comprehensive MDS-UPDRS-III score and distinct scores for rigidity (3.3), bradykinesia (sum of 3.4–3.8 and 3.14 scores), and tremor (sum of 3.15–3.18 scores) (Table 2). Differences in kinematic variables between PD patients at T0 and HCs were analyzed using an unpaired *t*-test. To assess possible changes in kinematic variables in PD patients between the two different sessions and the various time points considered, we performed separate repeated measures (rm)ANOVA with the within factors “experimental session” (2 levels: L-DOPA, L-DOPA + OPC), “side” (2 levels: most affected, less affected), and “time point” (4 levels: T0, T1, T2, T3). Furthermore, to specifically evaluate whether OPC modified the severity of motor fluctuations across the different time points (T0–T3), we calculated the CV of the various kinematic parameters (SD_T0-T3_/MEAN_T0-T3_*100) for each session and body side. We named this parameter Fluctuation Index (FI), with higher values reflecting greater fluctuations over the four time points. Then, we applied separate rmANOVA for each parameter using the within-group factors “session” and “side.” Post hoc analyses were performed using *t*-tests with the Tukey HSD test applied to correct for multiple comparisons. FI was also measured for the MDS-UPDRS-III scores, and possible differences between sessions were tested using the Wilcoxon test. Spearman correlation coefficient was used to test the possible relationship between clinical demographic (age, disease duration and MDS-UPDRS-III score, OFF state) and kinematic variables. For this purpose, we calculated the ratio between the values recorded in the “L-DOPA + OPC” session and those obtained in the “L-DOPA” one (e.g., ratio movement velocity L-DOPA + OPC/L-DOPA, ratio FI L-DOPA + OPC/L-DOPA). Values were also averaged across time points and body sides. The level of significance was set at *p* < 0.05. Statistical analyses were carried out using Statistica (TIBCO Software, USA).

## Results

Treatment with OPC was well tolerated in all patients, and as expected, LEDD significantly increased in the L-DOPA + OCP session (*p* < 0.001). Among the initially enrolled 20 patients, two participants could not complete the study due to personal reasons, resulting in their exclusion as dropouts. Consequently, the final data analysis was performed on 18 PD patients (Table 1).

**Table 1:** Clinical demographic characteristics of patients

Sub	Age (Y)	Gender	Dis.Dur. (Y)	MDS-UPDRS			H&Y	MoCA	BDI-II	LEDD (S1)	LEDD (S2)
				II	III	IV					
1	73	M	12	11	54	4	2	24	8	1440	2040
2	69	F	5	12	50	12	2	22	14	300	400
3	63	F	10	10	39	10	2	29	13	550	775
4	68	M	12	29	67	14	3	24	18	1250	1750
5	74	M	12	23	68	8	2	25	4	1250	1650
6	74	M	6	14	52	6	2	29	14	700	1050
7	76	M	10	24	40	3	2	22	13	500	700
8	58	M	11	9	44	7	2	28	9	900	1300
9	69	M	10	8	19	4	2	26	3	660	910
10	54	M	8	18	48	11	2	27	15	735	985
11	81	F	21	29	55	13	2	28	17	600	900
12	71	F	6	13	43	8	2	25	11	713	963
13	79	M	6	9	47	3	2	30	0	700	1000
14	76	M	4	18	46	2	2	22	5	700	1000
15	62	M	5	12	39	10	2	28	18	575	775
16	60	M	6	16	38	11	2	26	8	935	1235
17	65	F	4	12	23	7	2	22	3	840	1090
18	59	F	14	8	35	7	2	30	10	450	600
Mean	68.4	-	9.2	15.2	44.8	7.8	2.0	25.9	10.2	766.6	1062.4
SD	7.8	-	4.4	6.8	12.6	3.6	0.2	2.8	5.5	297.2	411.8

*Sub.* subject; *Y* years; *M* male; *F* female; *Dis.Dur.* disease duration; *MDS-UPDRS* Movement Disorder Society-Sponsored Revision of the Unified Parkinson’s Disease Rating Scale; *H&Y* Hoehn and Yahr scale; *MoCA* Montreal Cognitive Assessment; *BDI-II* Beck Depression Inventory II; *LEDD (S1)* L-DOPA equivalent daily dose in the first session (L-DOPA); *LEDD (S2)* L-DOPA equivalent daily dose in the second session (L-DOPA + OPC); *SD* standard deviation. LEDD are calculated according to previous literature data [37, 38]. Clinical scores reflect the values recorded before the L-DOPA intake (T0) in the L-DOPA session (i.e., without Opicapone)

### PD patients (OFF condition, L-DOPA session) vs HCs

**Fig. 2:**
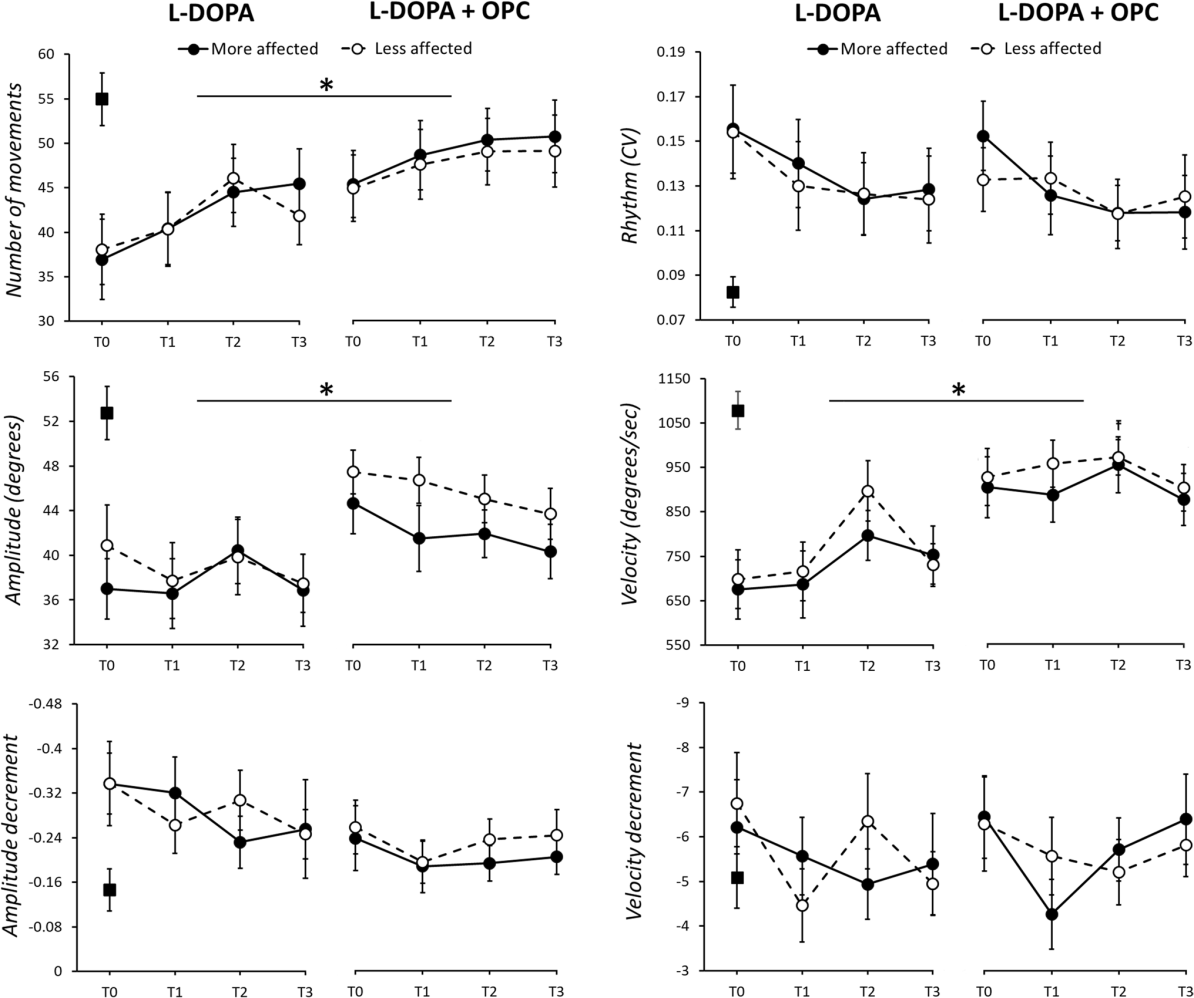
Effect of opicapone (OPC) on finger tapping kinematics. Black and white circles indicate mean values recorded in PD patients from the more and the less affected hand, in four distinct time points. Data from healthy controls (HCs) are also shown as black squares. Error bars denote standard errors. Asterisks indicate significant *p* values

Age (*p* = 0.56), gender distribution (*p* = 1.0), MoCA (*p* = 0.21), and BDI (*p* = 0.08) were comparable between PD patients and HCs. The kinematic analysis demonstrated that PD patients showed reduced velocity and amplitude (*p* < 0.001), a lower number of movements (*p* = 0.001), higher CV (less regular movement) (*p* < 0.001), and greater amplitude decrement compared to HCs (*p* = 0.01). Velocity decrement did not differ between groups (*p* = 0.37) (Fig. 2).

### Effects of Opicapone on clinical scale scores

MoCA (*p* = 0.81), BDI-II (*p* = 0.74), and MDS-UPDRS-II (*p* = 0.18) were similar between L-DOPA and L-DOPA + OPC sessions. Conversely, MDS-UPDRS-IV was lower in the L-DOPA + OPC than L-DOPA session (*p* < 0.01), suggesting less severe motor complications following the chronic intake of OPC.

When analyzing the clinical effect of OPC on motor symptoms severity, we observed significant changes in MDS-UPDRS-III scores, encompassing both total scores and subscores for rigidity, bradykinesia, and tremor across various time points in both sessions (L-DOPA, *p* < 0.001; L-DOPA + OPC, *p* < 0.001 for all variables). Notably, higher values were noted at T0 compared to other time points, while lower scores were evident at T2 compared to T1 and T3 (Table 2). Importantly, we consistently identified lower MDS-UPDRS-III scores in the L-DOPA + OPC session compared to the L-DOPA session at all tested time points (T0 and T3, *p* < 0.001; T1 and T2, *p* = 0.001). This pattern persisted when examining specific subscores dedicated to rigidity (T0, *p* = 0.02; T1, T2, and T3, *p* < 0.01), bradykinesia (*p* < 0.01 for all time points), and tremor (T0, *p* = 0.02; T1, *p* = 0.05; T2, *p* < 0.01; T3, *p* = 0.03) between sessions. Additionally, the FI calculated based on the MDS-UPDRS-III demonstrated comparable values between sessions (total score, *p* = 0.58; rigidity, *p* = 0.26; bradykinesia, *p* = 0.09; tremor, *p* = 0.10).

**Table 2:** MDS-UPDRS-III results and statistics

	L-DOPA session						L-DOPA + OPC session					
	Score	Statistics (Wilcoxon test)					Score	Statistics (Wilcoxon test)				
	*Mean (SD)*		*T0*	*T1*	*T2*	*T3*	*Mean (SD)*		*T0*	*T1*	*T2*	*T3*
TOT R B T	44.9 (12.4) 8.7 (3.8) 18.8 (6.1) 8.8 (4.3)	*T0*	-	** < 0.001** **0.02** **0.001** ** < 0.01**	** < 0.001** ** < 0.001** ** < 0.001** **0.001**	** < 0.01** 0.09 ** < 0.01** **0.03**	35.9 (9.7) 7.4 (4.1) 15.8 (5.7) 7.0 (3.7)	*T0*	-	** < 0.001** ** < 0.01** ** < 0.01** ** < 0.001**	** < 0.001** ** < 0.001** ** < 0.001** ** < 0.001**	** < 0.001** **0.01** ** < 0.01** **0.001**
TOT R B T	36.4 (11.4) 7.7 (3.8) 15.2 (4.8) 6.2 (5.0)	*T1*	** < 0.001** **0.02** **0.001** ** < 0.01**	-	** < 0.001** ** < 0.01** ** < 0.01** **0.02**	0.48 0.76 0.27 0.83	28.4 (8.8) 6.1 (3.2) 12.6 (5.2) 4.4 (3.7)	*T1*	** < 0.001** ** < 0.01** ** < 0.01** ** < 0.001**	-	** < 0.001** **0.001** ** < 0.01** **0.05**	0.92 0.86 0.91 0.46
TOT R B T	29.7 (10.8) 6.3 (3.4) 13.0 (5.6) 4.9 (4.2)	*T2*	** < 0.001** ** < 0.001** ** < 0.001** **0.001**	** < 0.001** ** < 0.01** ** < 0.01** **0.02**	-	** < 0.001** **0.01** ** < 0.001** **0.04**	23.7 (6.7) 4.8 (3.2) 9.4 (4.4) 3.4 (2.9)	*T2*	** < 0.001** ** < 0.001** ** < 0.001** ** < 0.001**	** < 0.001** **0.001** ** < 0.01** **0.05**	-	** < 0.001** **0.01** **0.001** 0.08
TOT R B T	37.8 (13.8) 7.7 (4.0) 16.1 (6.6) 6.6 (5.6)	*T3*	** < 0.01** 0.09 ** < 0.01** **0.03**	0.48 0.76 0.27 0.83	** < 0.001** **0.01** ** < 0.001** **0.04**	-	28.6 (9.0) 6.1 (3.5) 12.4 (4.2) 4.3 (3.3)	*T3*	** < 0.001** **0.01** ** < 0.01** **0.001**	0.92 0.86 0.91 0.46	** < 0.001** **0.01** **0.001** 0.08	-

The table shows the MDS-UPDRS-III total score (TOT), rigidity (R), bradykinesia (B), and tremor (T) subscores at all the time points tested (T0, T1, T2, and T3) in the two different experimental sessions (L-DOPA and L-DOPA + OPC) and the *p* values resulting from the non-parametric statistical comparisons between T0, T1, T2, and T3 scores. Significant *p* values are displayed in bold. *SD*, 1 standard deviation from the mean

### Effects of Opicapone on kinematic variables

When analyzing in detail the effect of OPC on bradykinesia through comprehensive kinematic assessment, we found that patients performed a higher number of movements (“experimental session,” *F*_1,17_ = 6.81, *p* = 0.02) and showed higher movement amplitude (“experimental session,” *F*_1,17_ = 5.22, *p* = 0.03) in the L-DOPA + OPC than the L-DOPA session independently from the time point considered and the body side (Table 3 and Fig. 2). Also, movement velocity was higher in the L-DOPA + OPC than the L-DOPA session (“experimental session,” *F*_1,17_ = 16.50, *p* < 0.001). This effect was prominent at specific time points and was present in both body sides (“experimental session” × “time point,” *F*_3,51_ = 2.87, *p* = 0.04) (Table 3). Post hoc analyses demonstrated a significantly higher movement velocity in the L-DOPA + OPC session at all the time points tested compared to the L-DOPA session, but this effect was prominent at T0, T1, and T3 (T0, T1, and T3, *p* < 0.001; T2, *p* = 0.02). Moreover, while movement velocity at T2 (L-DOPA peak dose) differed from T0 (*p* < 0.001), T1 (*p* = 0.001), and T3 (*p* = 0.04) in the L-DOPA session, T2 values were comparable to the other time points in the L-DOPA + OPC session (T2 vs T0, *p* = 0.83; T2 vs T1, *p* = 0.91; T2 vs T3, *p* = 0.34). Finally, the number of movements and movement velocity changed across the different time points tested (significant factor “time point”), with generally higher values at T2 than T0 (*p* < 0.01 for both parameters). Movement rhythm and amplitude and velocity decrement (sequence effect) did not change between the different sessions, time points, and body sides (see Table 3 for comprehensive statistical data). The values of kinematic parameters recorded at all the time points from the most and less affected body side in the two different experimental sessions are shown in the Supplementary Table 1.

**Table 3:** Results of the rmANOVAs

		rmANOVA, raw data			rmANOVA, FI	
Parameter		Main factors		Interaction terms		Main factors
N. Mov	**E**	***F***_**1,17**_** = 6.81, *p* = 0.02**	**ES**	*F*_1,17_ = 0.80, *p* = 0.38	**E**	***F***_**1,17**_** = 10.42, *p* < 0.01**
	**S**	*F*_1,17_ = 0.99, *p* = 0.33	**ET**	*F*_3,51_ = 1.15, *p* = 0.34	**S**	*F*_1,17_ = 1.97, *p* = 0.18
	**T**	***F***_**3,51**_** = 7.16, *p* < 0.001**	**ST**	*F*_3,51_ = 2.11, *p* = 0.11	**T**	***F***_**1,17**_** = 1.12, *p* = 0.30**
			**EST**	*F*_3,51_ = 1.25, *p* = 0.30		
Vel	**E**	***F***_**1,17**_** = 16.50, *p* < 0.001**	**ES**	*F*_1,17_ = 0.01, *p* = 0.97	**E**	***F***_**1,17**_** = 7.68, *p* = 0.01**
	**S**	*F*_1,17_ = 2.18, *p* = 0.16	**ET**	***F***_**3,51**_** = 2.87, *p* = 0.04**	**S**	*F*_1,17_ = 0.05, *p* = 0.82
	**T**	***F***_**3,51**_** = 5.62, *p* < 0.01**	**ST**	*F*_3,51_ = 1.23, *p* = 0.31	**T**	*F*_1,17_ = 1.01, *p* = 0.33
			**EST**	*F*_3,51_ = 2.28, *p* = 0.09		
Amp	**E**	***F***_**1,17**_** = 5.22, *p* = 0.03**	**ES**	*F*_1,17_ = 1.81, *p* = 0.20	**E**	***F***_**1,17**_** = 5.97, *p* = 0.02**
	**S**	*F*_1,17_ = 1.74, *p* = 0.20	**ET**	*F*_3,51_ = 1.49, *p* = 0.23	**S**	*F*_1,17_ = 3.09, *p* = 0.10
	**T**	*F*_3,51_ = 1.38, *p* = 0.26	**ST**	*F*_3,51_ = 0.91, *p* = 0.44	**T**	*F*_1,17_ = 0.01, *p* = 0.91
			**EST**	*F*_1,17_ = 1.81, *p* = 0.20		
CV	**E**	*F*_1,17_ = 0.26, *p* = 0.61	**ES**	*F*_1,17_ = 0.06, *p* = 0.80	**E**	*F*_1,17_ = 0.20, *p* = 0.65
	**S**	*F*_1,17_ = 0.09, *p* = 0.77	**ET**	*F*_3,51_ = 0.07, *p* = 0.98	**S**	*F*_1,17_ = 0.07, *p* = 0.79
	**T**	*F*_3,51_ = 2.06, *p* = 0.12	**ST**	*F*_3,51_ = 0.50, *p* = 0.68	**T**	*F*_1,17_ = 0.13, *p* = 0.71
			**EST**	*F*_1,17_ = 0.06, *p* = 0.80		
Amp.Dec	**E**	*F*_1,17_ = 3.38, *p* = 0.08	**ES**	*F*_1,17_ = 0.26, *p* = 0.61	**E**	*F*_1,17_ = 0.55, *p* = 0.47
	**S**	*F*_1,17_ = 0.26, *p* = 0.61	**ET**	*F*_3,51_ = 1.56, *p* = 0.21	**S**	*F*_1,17_ = 0.15, *p* = 0.70
	**T**	*F*_3,51_ = 1.61, *p* = 0.20	**ST**	*F*_3,51_ = 0.77, *p* = 0.52	**T**	*F*_1,17_ = 0.12, *p* = 0.73
			**EST**	*F*_3,51_ = 0.31, *p* = 0.82		
Vel.Dec	**E**	*F*_1,17_ = 0.24, *p* = 0.63	**ES**	*F*_1,17_ = 0.02, *p* = 0.90	**E**	***F***_**1,17**_** = 5.28, *p* = 0.03**
	**S**	*F*_1,17_ = 0.01, *p* = 0.92	**ET**	*F*_3,51_ = 0.67, *p* = 0.57	**S**	*F*_1,17_ = 0.01, *p* = 0.93
	**T**	*F*_3,51_ = 2.59, *p* = 0.06	**ST**	*F*_3,51_ = 0.46, *p* = 0.71	**T**	*F*_1,17_ = 0.10, *p* = 0.75
			**EST**	*F*_3,51_ = 2.47, *p* = 0.07		

*rmANOVA* repetitive measures analysis of variance; *FI* Fluctuation Index; *N.Mov* number of movements; *Vel* velocity; *Amp* amplitude; *CV* coefficient of variation; *Amp.Dec.* amplitude decrement; *Vel.Dec.* velocity decrement; *E* experimental session; *S* side; *T* time point; *ES* “experimental session” × “side”; *ET* “experimental session” × “time point”; *ST* “side” × “time point”; *EST* “experimental session” × “side” × “time point”; experimental session levels, L-DOPA, L-DOPA + OPC; *side levels* most affected, less affected; time point levels, T0, T1, T2, and T3 Significant factors and interactions are displayed in bold

**Fig. 3:**
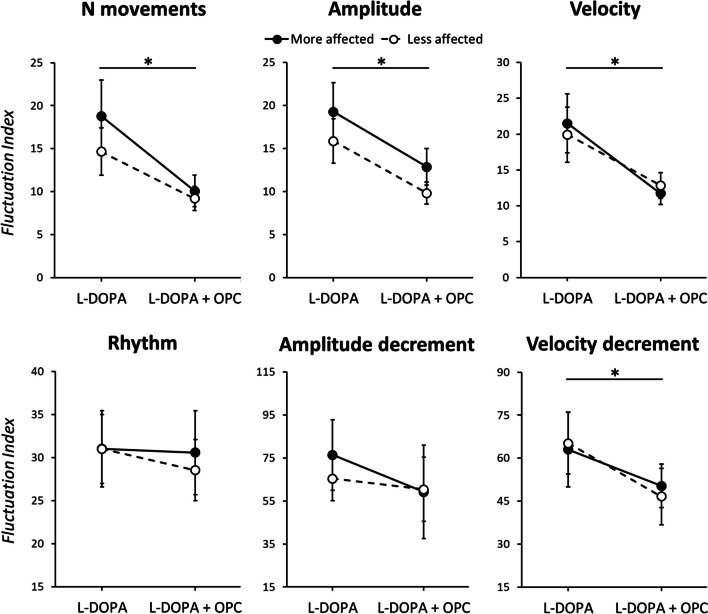
Impact of opicapone (OPC) on the severity of bradykinesia fluctuations in Parkinson’s disease (PD) patients. The Fluctuation Index (FI) across the different time points (T0–T3) for each kinematic parameter in the two sessions is shown. Higher FI values reflect greater fluctuations over the four time points. Black and white circles indicate values recorded in patients from the more and the less affected hand, respectively. Error bars denote standard errors. Asterisks indicate significant *p* values

Importantly, the specific analysis conducted to assess the impact of OPC on the severity of bradykinesia fluctuations across different time points provided further insights. We found lower FI in the L-DOPA + OPC session compared to the L-DOPA session for several kinematic parameters, including the number of performed movements (“experimental session,” *F*_1,17_ = 10.42, *p* < 0.01), movement velocity (“experimental session,” F_1,17_ = 7.68, *p* = 0.01), and amplitude (“experimental session,” *F*_1,17_ = 5.97, *p* = 0.02). The observed effect was consistent across both body sides and did not manifest when analyzing movement rhythm and amplitude decrement (see Table 3 and Fig. 3).

### Clinical–neurophysiological correlations

The correlation analysis disclosed no significant relationship between the patient’s age, disease duration, or motor symptom severity at baseline (i.e., MDS-UPDRS-III at T0, L-DOPA session) and the effects of OPC on movement kinematics and their FIs (age vs. effects of OPC, *r* ranging from − 0.27 to 0.15, *p* ranging from 0.27 to 0.86; disease duration vs. effects of OPC, *r* ranging from − 0.20 to 0.43, *p* ranging from 0.07 to 0.65; MDS-UPDRS-III vs. effects of OPC, *r* ranging from − 0.45 to 0.41, *p* ranging from 0.07 to 0.55).

## Discussion

In our current study, we performed a comprehensive kinematic analysis and demonstrated that adding OPC to the standard dopaminergic therapy in PD patients improves various movement parameters and reduces bradykinesia fluctuations after single-dose L-DOPA intake. The effects were prominent at specific time points attributed to relevant periods associated with L-DOPA dynamics. Even if we did not measure L-DOPA pharmacokinetic, our results can be interpreted as an effect of OPC on peripheral L-DOPA metabolism [25]. More generally, our findings extend observations on the use of objective motion kinematic analysis techniques to investigate the effects of PD drugs on bradykinesia.

In considering our results, we excluded certain potential confounding factors. Firstly, we ruled out influences related to demographic factors (such as age and gender distribution) and the impact of cognitive deficits and other neuropsychiatric disorders, since these variables were comparable to those observed in the control subjects. Additionally, all patients were studied within the same time frame, starting from the morning, thus ruling out the potential influence of circadian factors. The dopaminergic therapy of patients remained constant between the two sessions, except for the addition of OPC. Consequently, our findings can be attributed solely to the effects of OPC. Finally, as patients were encouraged to perform to the best of their abilities during each experiment and were provided with rest breaks, we excluded the possibility of motor performance being influenced by non-specific factors, including possible attention fluctuations and fatigue.

The novel aspect of the study is the objective assessment of the effects of OPC on bradykinesia through kinematic analysis instead of the clinical examination alone, which can be influenced by potential evaluation-dependent bias as well as a certain degree of approximation [26, 27]. The kinematic analysis provides a precise and quantitative evaluation of movement changes, particularly velocity and amplitude, allowing for a more in-depth documentation of the effects of OPC on motor function in patients. This issue is particularly relevant, given the clinical discrepancies observed in previous studies [28]. These discrepancies may arise from subjective improvements that patients appreciate but are not captured by standard rating scales or recognized by clinicians [28]. Interestingly, we first observed that the greatest benefits of OPC were observed in the OFF phase, indicating that OPC significantly improved the early morning-OFF phenomenon (baseline/T0). Second, because at T1 the OPC session showed visibly higher kinematic parameters than the session without OPC, our results also suggest a probable effect of OPC on the delayed-ON phenomenon. These results support the concept of a more general benefit of OPC on PD mobility beyond well-established extension of ON time [29]. Finally, at T2 and T3, we observed a slower decline in kinematic parameters after OPC, thus indicating a possible benefit on the wearing-OFF phenomenon compared to the session without OPC. Another essential finding of our study was the reduction in measurement variability after OPC intake. These results may suggest a more stable movement velocity across the various time points in the L-DOPA + OPC session as also demonstrated by the analysis of the FI. The FI, which reflected the coefficient of variation of kinematic parameters across the different time points, was indeed significantly lower in the L-DOPA + OPC compared to the L-DOPA session for several kinematic parameters, including movement velocity and amplitude. This reduction in variability implies a more stable and consistent response to treatment, suggesting that OPC plays a crucial role in improving the predictability and effectiveness of L-DOPA therapy. Overall, our data confirm the observations of previous clinical studies [3, 4, 30] demonstrating that OPC can attenuate motor symptoms and motor fluctuations in PD. Our findings also align with recent pharmacokinetic/pharmacodynamics plasma dosage research, which offers pharmacological evidence supporting OPC’s effects [25, 31]. As known from previous studies, OPC can stabilize the mean concentration of L-DOPA in the blood and prolong its effects. Moreover, OPC administration may contribute to achieving a higher maximum plasma concentration of L-DOPA [32, 33], thus prolonging the effects of the last evening dose of L-DOPA [31, 34, 35]. In addition to the pharmacokinetic effects of OPC, it is also possible that the administration of this medication significantly contributed, alongside L-DOPA, to the normalization of some neurophysiological dysfunctions that play a key role in bradykinesia pathophysiology [8]. These abnormalities may include motor cortex disinhibition, impaired plasticity mechanisms, and altered oscillatory activities within the basal ganglia–thalamo–cortical circuits [8, 12, 20, 36–38]. Investigating these aspects was beyond the scope of the current study; however, we believe that further exploration of the neurophysiological effects of OPC should be specifically examined in future studies.

Other experimental studies have employed motion kinematic analysis techniques to investigate the effects of dopaminergic drugs on motor performance in PD [8]. However, only a few have specifically examined finger tapping, the most sensitive test commonly used in clinical practice to assess the effects of medication on bradykinesia, and these include L-DOPA, selegiline, and jejunal L-DOPA infusion [6, 7, 11, 24]. Consistent with previous observations on other medications, we have also observed that the administration of OPC predominantly improved movement velocity [11], the most sensitive parameter that can be modified after dopaminergic drug administration. In the present study, we also observed that OPC administration significantly improved movement amplitude. However, the OPC effect on amplitude was overall lower than the effect observed on velocity. We found no benefit of OPC for movement rhythm and amplitude decrement after its administration, confirming that these bradykinesia components are characterized by distinct pathophysiological mechanisms [8, 13]. Finally, consistent with previous studies [7, 12, 20, 36], velocity decrement demonstrated no significant differences between PD patients and HC. This observation possibly also explains the lack of changes after OPC intake. Notably, the neurophysiologic assessment of the effects of OPC in advanced PD patients demonstrates an improvement, although not normalization, of motor abnormalities.

The main limitation encountered in this study was the relatively small sample size. However, the objective nature of kinematic analysis ensures reliable results even with small sample sizes [39]. Another possible limitation is that the patients were not blinded to the administration of OPC, thus implying possible placebo effects. Despite the lack of blinding in our study, the pattern of improvement in specific kinematic variables, namely, movement velocity and amplitude, aligns substantially with previous observations. We believe that the improvement of these specific parameters provides additional support to the notion that the observed effects are genuinely related to the improvement of L-DOPA pharmacokinetic after OPC administration and not significantly influenced by placebo responses. Additionally, it is important to note that our study had a relatively short follow-up period, and as a result, we cannot ascertain how long the short-term positive effects of OPC on movement parameters will persist. We know the effect OPC occurs already after a few days after therapy initiation, but to gain a comprehensive understanding of the lasting benefits of OPC on kinematic measurements, further evaluations with longer follow-up durations will be necessary. However, a longitudinal study would have to consider the progressive worsening of motor disability and the need to further adjust concomitant therapy. Finally, it is important to note that in our study we have only investigated the interactions between OPC and L-DOPA in patients with advanced disease, while OPC benefit may be even greater in early fluctuators on limited doses of L-DOPA or in stable patients [5, 40].

## Conclusion

The data of this study demonstrated that adding OPC improves bradykinesia in PD patients by increasing the velocity and amplitude of movements performed, in both ON and morning-OFF and end-of-dose phases. We have shown that the greatest benefits are present at different times, i.e., upon awakening (morning-OFF phenomenon) as well as during end-of-dose deterioration (wearing-off phenomenon). In the latter case, we can infer that OPC is responsible for a much more gradual decline in the effect of L-DOPA. The results confirm and extend previous observations obtained in earlier studies concerning the pharmacokinetics of OPC and those of the main clinical trials conducted so far. The study’s results also allowed us to obtain relevant information regarding the pharmacological effects of OPC on movement in PD and suggest a number of further studies that could be conducted, in particular the longitudinal evaluation of effects of OPC in stable and early fluctuator patients.

### Supplementary Information

Below is the link to the electronic supplementary material.

Supplementary file1 (DOCX 17 KB)

## Data Availability

The datasets generated and analyzed during the current study are available from the corresponding author on reasonable request.

## Abbreviations

ANOVA: Analysis of variance
BDI: Beck Depression Inventory
CV: Coefficient of variation
COMT: Catechol O-methyl transferase
FI: Fluctuation Index
HC: Healthy control
MoCA: Montreal Cognitive Assessment
MDS-UPDRS: Movement Disorder Society-Sponsored Revision of the Unified Parkinson’s Disease Rating Scale
OPC: Opicapone
PD: Parkinson’s disease
SD: Standard deviation
