# Correction to: Objective assessment of the effects of opicapone in Parkinson’s disease through kinematic analysis

**DOI:** 10.1007/s10072-023-07294-7

**Published:** 2023-12-28

**Authors:** Matteo Bologna, Andrea Guerra, Donato Colella, Daniele Birreci, Davide Costa, Antonio Cannavacciuolo, Luca Angelini, Giulia Paparella, Angelo Antonini, Alfredo Berardelli, Giovanni Fabbrini

**Affiliations:** 1 Department of Human Neurosciences, Sapienza University of Rome, Viale Dell’Università 30, 00185 Rome, Italy; 2 IRCCS Neuromed, 86077 Pozzilli, IS Italy; 3 Parkinson and Movement Disorder Unit, Study Center On Neurodegeneration (CESNE), Department of Neuroscience, University of Padua, Padua, Italy; 4 Present Address: Padua Neuroscience Center, University of Padua, Padua, Italy

**Correction to: Neurological Sciences (2023)**

10.1007/s10072-023-07233-6

The originally published article contains an error. In Table 3, author noticed that there are some values that for some reasons have been incorporated in the Table during the proofreading whereas they should be removed. This is under the column of “Interaction terms”.

The original article has been corrected.

