# Supplementary material for: Objective assessment of the effects of opicapone in Parkinson’s disease through kinematic analysis
Source: Neurol Sci. 2023 Dec 13;45(5):2035–46. doi: 10.1007/s10072-023-07233-6 (PMC11021230; doi:10.1007/s10072-023-07233-6)
Supplement: Supplementary file 1 — Supplementary file1 (DOCX 17 KB) [file 10072_2023_7233_MOESM1_ESM.docx]

**Supplementary Table 1. Kinematic data**

|  | **L-DOPA SESSION** | | | | | | | | |  | **L-DOPA + OCP SESSION** | | | | | | | |
| --- | --- | --- | --- | --- | --- | --- | --- | --- | --- | --- | --- | --- | --- | --- | --- | --- | --- | --- |
|  | **most affected side** | | | | | **less affected side** | | | |  | **most affected side** | | | | **less affected side** | | | |
|  | *T0* | *T1* | *T2* | *T3* | *T0* | | *T1* | *T2* | *T3* |  | *T0* | *T1* | *T2* | *T3* | *T0* | *T1* | *T2* | *T3* |
| **N. Mov** | 36.9 (19.1) | 40.4  (17.2) | 44.5 (16.3) | 45.5 (16.5) | 38.1 (16.7) | | 40.4 (17.7) | 46.1 (16.1) | 41.9 (13.9) |  | 45.7 (16.4) | 49.1 (16.9) | 50.8 (15.4) | 51.2 (17.8) | 45.2 (16.3) | 48.0 (17.1) | 49.5 (16.2) | 49.5 (17.7) |
| **Vel** | 675 (282) | 686 (320) | 796 (238) | 753 (277) | 698 (278) | | 716 (280) | 897 (290) | 730 (204) |  | 903 (290) | 886 (262) | 953 (268) | 875 (250) | 925 (272) | 956 (225) | 970 (168) | 901 (221) |
| **Amp** | 37.0 (11.4) | 36.6 (13.2) | 40.4 (12.6) | 36.9 (13.6) | 40.9 (15.4) | | 37.7 (14.4) | 39.8 (14.3) | 37.5 (11.1) |  | 44.7 (11.6) | 41.5 (12.6) | 41.9 (9.1) | 40.3 (10.2) | 47.5 (8.3) | 46.7 (8.8) | 45.0 (9.0) | 43.7 (9.7) |
| **CV** | 0.15 (0.08) | 0.14 (0.08) | 0.12 (0.07) | 0.13 (0.08) | 0.15 (0.09) | | 0.13 (0.08) | 0.13 (0.08) | 0.12 (0.08) |  | 0.15 (0.07) | 0.13 (0.08) | 0.12 (0.05) | 0.12 (0.07) | 0.13 (0.06) | 0.13 (0.07) | 0.12 (0.07) | 0.13 (0.08) |
| **Amp.Dec.** | -0.34 (0.23) | -0.32 (0.27) | -0.23 (0.20) | -0.25 (0.37) | -0.34 (0.32) | | -0.26 (0.22) | -0.31 (0.23) | -0.25 (0.19) |  | -0.24 (0.25) | -0.19 (0.21) | -0.20 (0.14) | -0.21 (0.14) | -0.26 (0.21) | -0.20 (0.16) | -0.24 (0.16) | -0.25 (0.20) |
| **Vel.Dec.** | -6.21 (4.50) | -5.57 (3.68) | -4.93 (3.34) | -5.39 (4.81) | -6.75 (4.82) | | -4.46 (3.48) | -6.35 (4.52) | -4.95 (3.01) |  | -6.54 (4.05) | -4.30 (3.44) | -5.80 (3.11) | -6.50 (4.42) | -6.39 (4.61) | -5.65 (3.79) | -5.28 (3.20) | -5.89 (3.06) |

Values reflect the mean (1 standard deviation from the mean) of kinematic parameters recorded at all the time points from the most and less affected body side in the two different experimental sessions. N.Mov: number of movements; Vel: velocity; Amp: amplitude; CV: Coefficient of Variation; Amp.Dec.: amplitude decrement; Vel.Dec.: velocity decrement
